# StatiCAL: an interactive tool for statistical analysis of biomedical data and scientific valorization

**DOI:** 10.1186/s12859-024-05829-z

**Published:** 2024-06-12

**Authors:** Tanguy Pace-Loscos, Jocelyn Gal, Sara Contu, Renaud Schiappa, Emmanuel Chamorey, Dorian Culié

**Affiliations:** 1 Département d’Epidémiologie, de Biostatistique et des Données de Santé, Centre Antoine Lacassagne, Université Cote d’Azur, Nice, France; 2 Institut Universitaire de la Face et du Cou, Centre Antoine Lacassagne, Département de Chirurgie Cervico-Faciale, Université Cote d’Azur, 06103 Nice, France

**Keywords:** Medical statistics, Data analysis, Statistical software, Data management

## Abstract

**Background:**

In the realm of biomedical research, the growing volume, diversity and quantity of data has escalated the demand for statistical analysis as it is indispensable for synthesizing, interpreting, and publishing data. Hence the need for accessible analysis tools drastically increased. StatiCAL emerges as a user-friendly solution, enabling researchers to conduct basic analyses without necessitating extensive programming expertise.

**Results:**

StatiCAL includes divers functionalities: data management, visualization on variables and statistical analysis. Data management functionalities allow the user to freely add or remove variables, to select sub-population and to visualise selected data to better perform the analysis. With this tool, users can freely perform statistical analysis such as descriptive, graphical, univariate, and multivariate analysis. All of this can be performed without the need to learn R coding as the software is a graphical user interface where all the action can be performed by clicking a button.

**Conclusions:**

StatiCAL represents a valuable contribution to the field of biomedical research. By being open-access and by providing an intuitive interface with robust features, StatiCAL allow researchers to gain autonomy in conducting their projects.

## Introduction

The computerization of patient records [1], the development of data warehouses in healthcare facilities [2], the availability of large national and international databases such as the Health Data Hub (https://www.health-data-hub.fr/ [3]) or the Cancer Genome Atlas Project (TCGA) (https://portal.gdc.cancer.gov/) [4], and new biology techniques like high-throughput sequencing [5] enable the access to increasingly numerous, voluminous, and comprehensive biomedical databases. Systematic recourse to statistical analyses is indispensable for synthesizing, interpreting, and publishing this data [6], and many scientific journals specify in their author guidelines the recommendations for statistical analyses and result presentation [7–11]. For example, the New England Journal of Medicine recommends detailed reporting of statistical methods, including the justification of sample size, methods for handling missing data, and the use of confidence intervals and exact *p* values [7].

The implementation of statistical analyses, both descriptive and inferential, is thus a crucial element in any research project. These analyses can be conducted using various tools such as R, which is open-source software (GNU license) [12] and widely utilized within the scientific community [13]. However, while R is powerful and flexible, it requires a significant time investment to learn, especially for complex data sets, and performing quality analyses often necessitates advanced programming knowledge, thereby limiting its usage to trained personnel only.

Our objective was to design and provide a user-friendly, interactive tool allowing users to conduct basic and essential statistical analyses autonomously without any programming knowledge.

## Materials and methods

### Implementation

StatiCAL was developed using the R programming language, utilizing the “shinydashboard” package, which extends the functionality of the “shiny” package to a dashboard format, and “shinyWidgets,” which adds widgets to those already available. Data file reading is done using the “readxl” package. The application utilizes basic R functions, the “ggplot2” package, and the “survival” package for survival analyses. The “prediction” package, in conjunction with reworked codes from the “DynNom” package [14], enables nomogram generation. The “gtsummary,” “finalfit,” and “flextable” packages are used to generate result tables from statistical analyses. The “DT” package allows for dynamic data display. The “officer” package allows the construction of automatic and aesthetically pleasing reports for presenting analysis results. StatiCAL is hosted on a shiny server [15] available on the internal network of the Centre Antoine Lacassagne. Access to the application is through a portal, eliminating the need for direct manipulation of R.

### Data

The analyzed file must be in Excel^®^ format and have 2 worksheets. The first one contains the data table with the variable names, and the second one provides, for each variable, its label (more descriptive than the name) and its format. A data formatting step is required before the user can begin the analyses using the tool. An example is available in the “Tutorial” section.

### Operation

The application is organized into distinct blocks, each operating from the initial dataset. Each block is independent and allows for the completion of a specific task. To facilitate the use of StatiCAL, the user manipulates the labels of variables, which are more understandable than the variable names. The program then matches the label with the variable name to retrieve the associated data for each label.

A version of the tool is available at the following address: https://debds.shinyapps.io/StatiCAL/.

## Results

The user accesses the functionalities of the tool, categorized into various tabs as described below.

### Tutorial

**Fig. 1:**
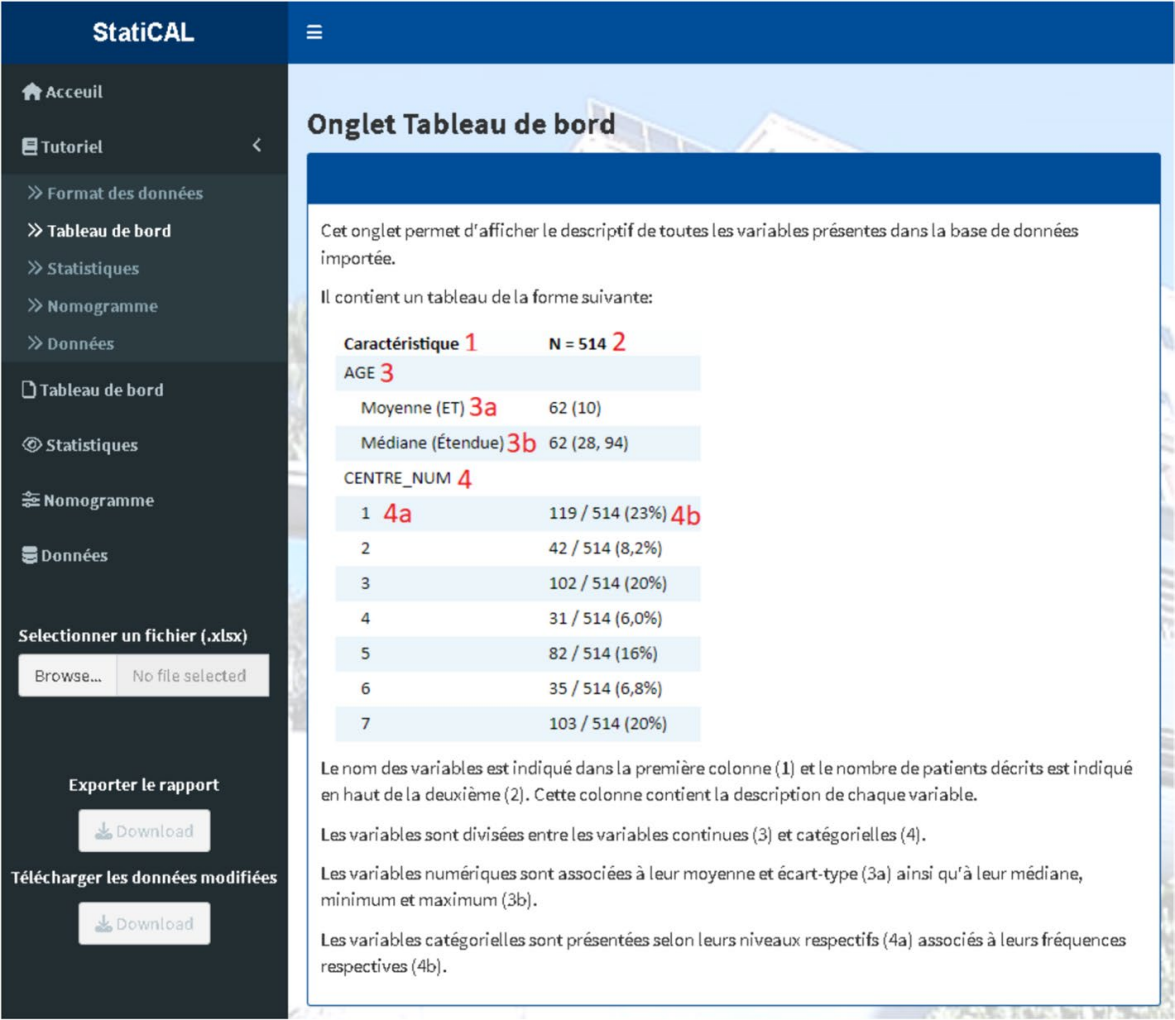
Dashboard tab tutorial

A user guide for StatiCAL is available in the Tutorial tab (Fig. 1), ensuring complete autonomy in using the tool even for users who are novices in statistical analysis. This tab allows for the description of the functionality of each feature.

### Data loading

**Fig. 2:**
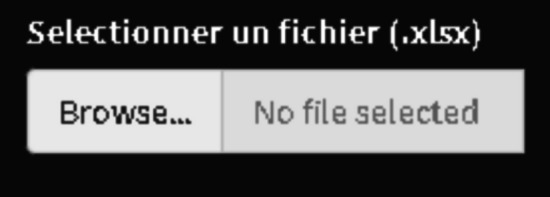
File upload button

The data is loaded via the file explorer of the current operating system, allowing direct loading of the file in Excel^®^ format (Fig. 2). Data formatting of the file must be done beforehand according to the detailed recommendations in the “Tutorial” section.

### Dashboard

**Fig. 3:**
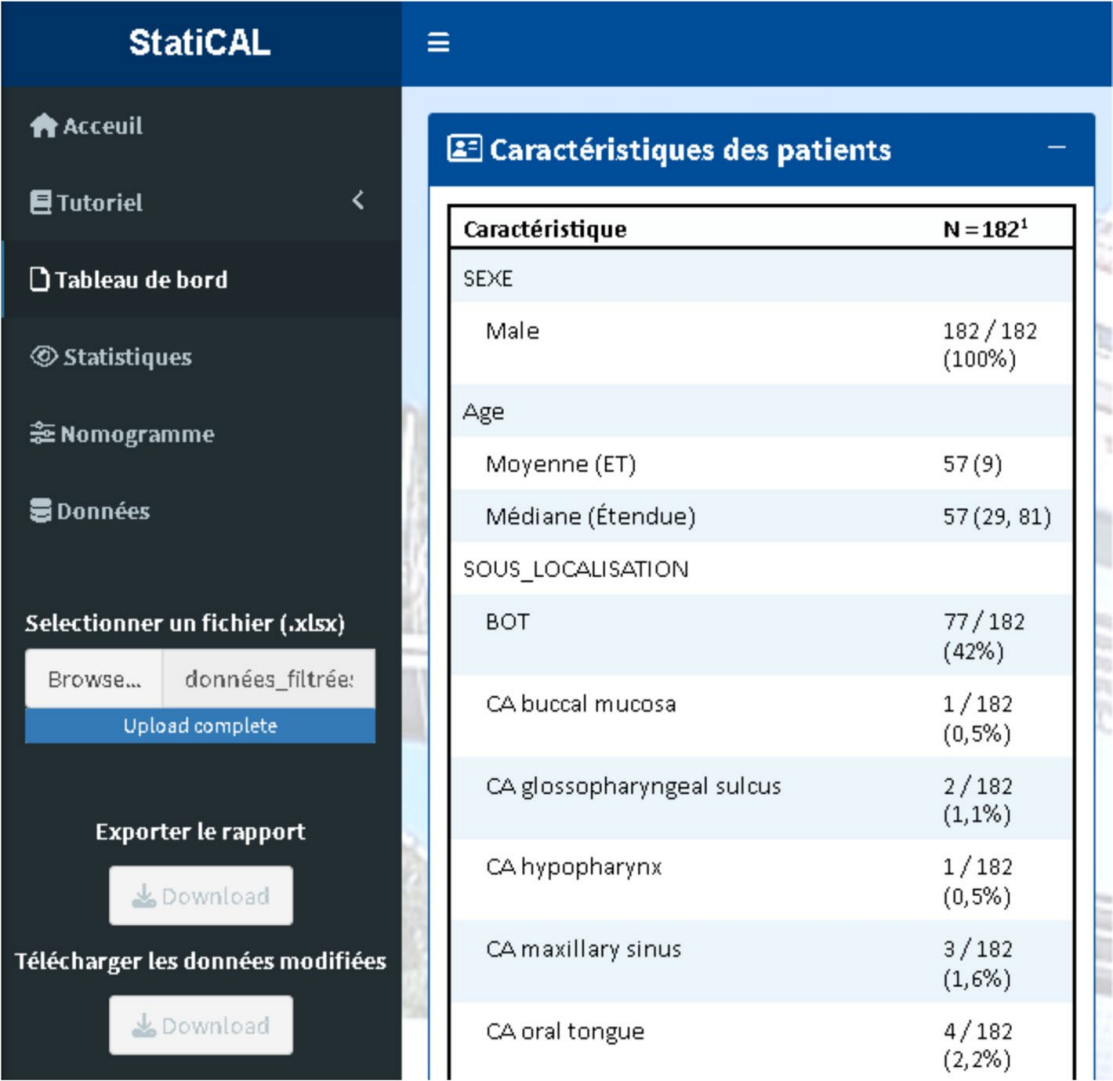
Dashboard tab

This section provides an overall description (Fig. 3) of all variables in the file. This step allows the user to perform a qualitative and quantitative check of the data contained in the dataset. It enables visualizing the number of missing data per variable and identifying potential outliers that need correction.

### Statistics

**Fig. 4:**
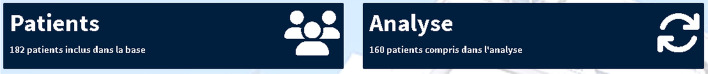
Indication of the number of patients in the dataset and in the analysis

This tab indicates the number of patients present in the initial database and the number of patients on which the analysis was conducted (Fig. 4). This number can be modified through the Data tab by selecting a sub-population.

**Fig. 5:**
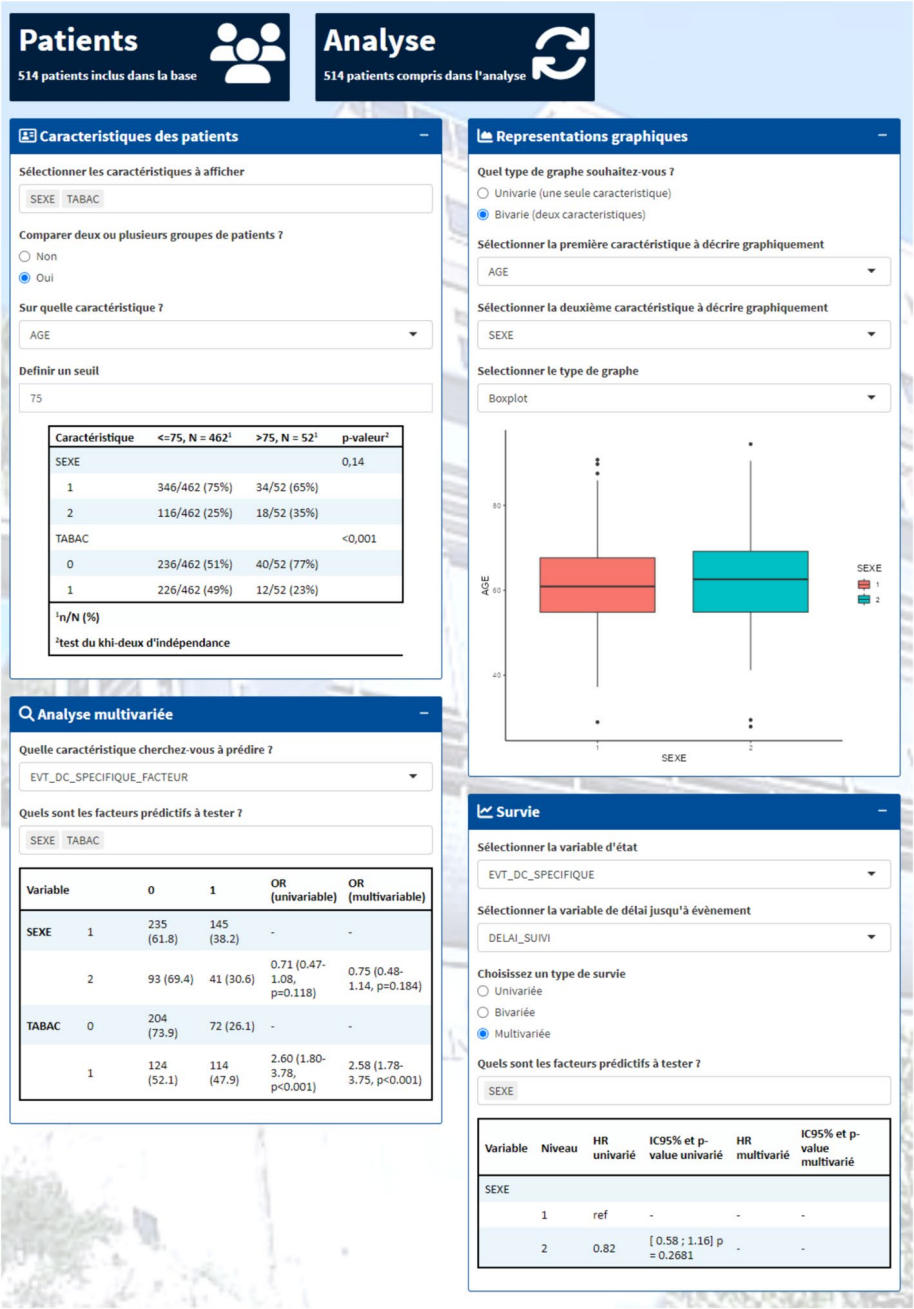
Statistics tab

This statistical section is divided into four different fields (Fig. 5), each presenting specific functionalities. The first functionality of this page is to display, based on the selected variables, patient characteristics, and perform bivariate analyses based on mean and/or proportion comparisons. A summary table presenting information according to the selected variable types is generated. Absolute and relative frequencies are presented for categorical variables, while mean, median, standard deviation, and range are provided for quantitative variables. In the case of bivariate analysis, the initial frequencies for each variable are distributed among the different levels of the comparison variable. If the comparison variable is numeric, the user is asked to choose a threshold to create 2 groups for comparison. The frequencies between the groups are then compared to conclude whether or not there is a statistical difference. The second functionality is to display univariate or bivariate graphical representations of the user-selected variables. Different types of graphs are available: boxplot, barplot, and histogram. The third functionality allows for the construction of logistic regression models, explaining a binary variable, such as response to treatment, based on several prognostic factors. The table presents the raw and adjusted odds ratios, both univariate and multivariate, of the model with their associated p-value for selecting the final model. The fourth functionality corresponds to survival analyses, both univariate, bivariate, and multivariate. Univariate and bivariate analyses are represented in the form of Kaplan-Meier curves, associated with survival median, follow-up median, and a summary table at certain time points containing the number of patients at risk, the number of events, the number of censored patients, the percentage of survivors, and its 95% confidence interval. In bivariate analysis, the different identified groups are compared, and the generated tables contain information for each group. In multivariate analysis, a single table is generated showing the Hazard Ratios (HR), both univariate and multivariate, with associated p-values to select the final model.

### Nomogram

**Fig. 6:**
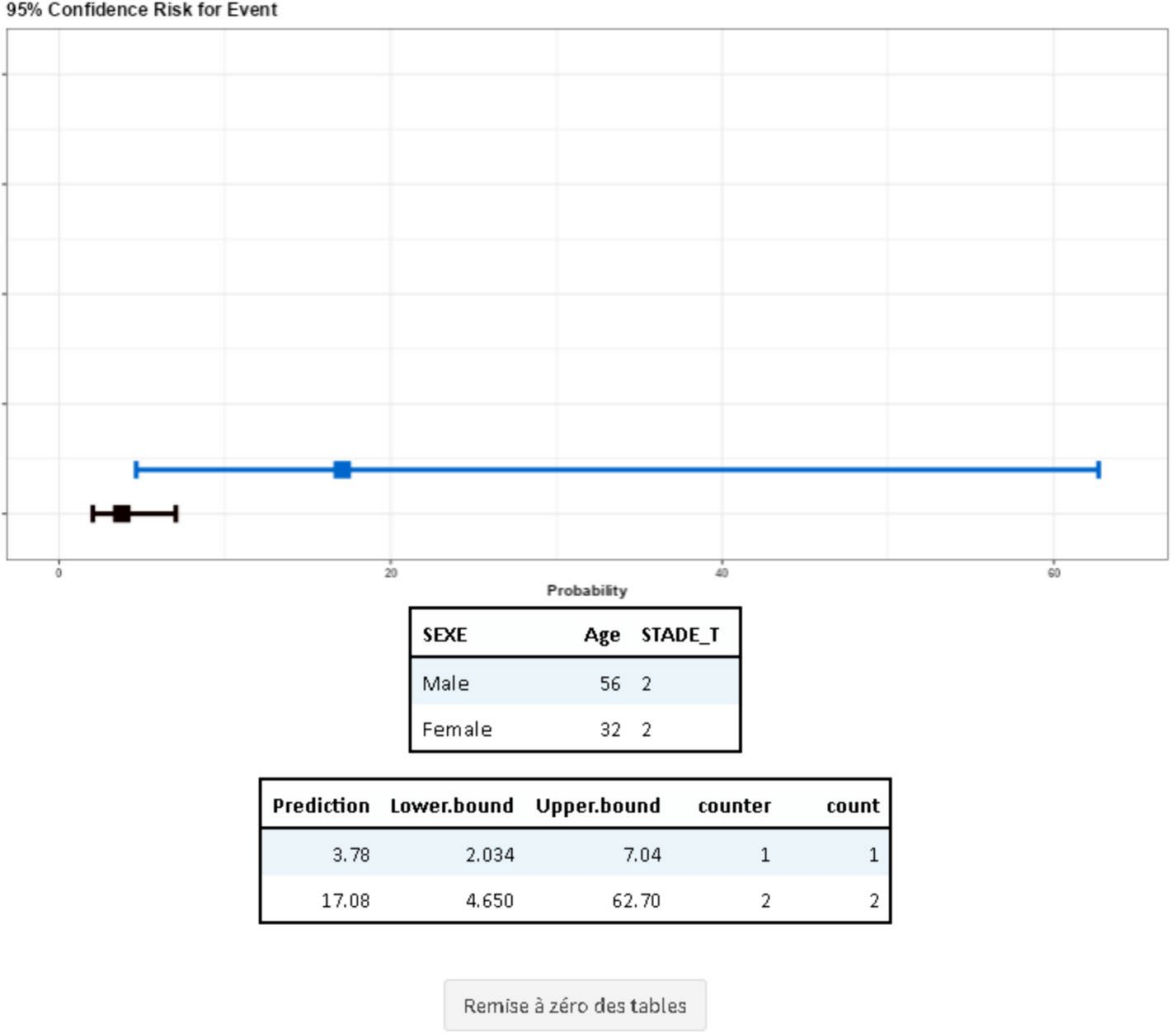
Example of a nomogram, risk of death based on sex, age, and T stage

The nomogram is a prediction tool. Based on a multivariate model constructed from the statistical analyses performed, it is possible to predict the risk for a patient to experience the event explained by the model (Fig. 6).

### Data

**Fig. 7:**
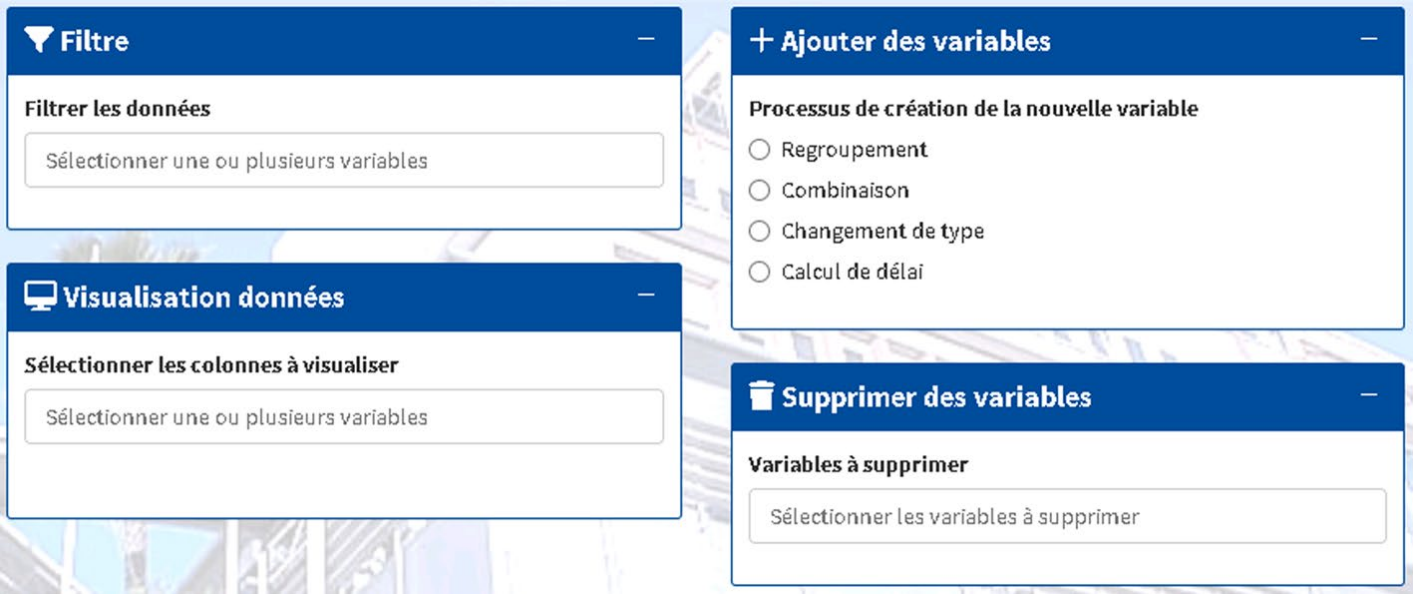
Data tab

This is a data manipulation tool. It has 4 functionalities (Fig. 7). The first one is to filter the imported data to create a subgroup for analysis. The second functionality allows for creating new variables from those present in the database. Different methods are available: by grouping levels of a variable, by combinations of variables to identify patients with common characteristics, by changing the type of a variable, and by calculating a delay from a date. These variables can be removed from the database, which constitutes the third functionality of this tab. The fourth functionality allows for visualizing the database to directly check the data present or created in the data file.

### Data export

**Fig. 8:**
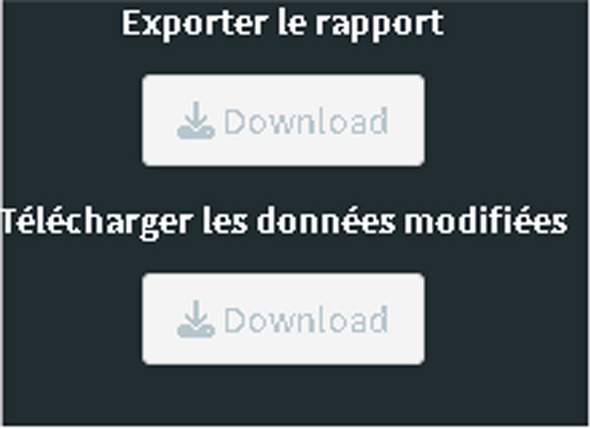
Export buttons

The analyses conducted in the Statistics tab are exportable in a editable Word^®^ format. The user can then directly retrieve the obtained results. The filtered and/or modified data are also exportable in Excel^®^ format to preserve the changes made (Fig. 8).

## Discussion

The StatiCAL tool is a free application developed by the Département de Biostatistique et des Données de Santé in collaboration with physicians and researchers from the Centre Antoine Lacassagne. It meets the initial objectives of enabling the conduct of simple statistical analyses by non-specialist personnel. It allows for various descriptive, graphical, and inferential statistical analyses regardless of the type of data studied (discrete or continuous quantitative variables, binary or multimodal qualitative variables, or censored data).

StatiCAL is intended for all professionals, whether physicians or researchers, who are faced with the analysis of biomedical data. While fostering collaboration between medical teams, research teams, and biostatistics teams is important, access to these resources is often difficult, leading to significant delays and requiring funding sources. Thus, StatiCAL represents an interesting tool that allows physicians and researchers to have some independence in their scientific project management and the resulting analyses. The tool appears easy to use, and the autonomy it provides is highly appreciated. It allows for a simple exploratory analysis before validating more complex models with a statistician, conducting simple analyses for a thesis or dissertation, or performing additional analyses to an initial analysis. As the functionalities used in StatiCAL are voluntarily limited to some analyses, the packages used do not require modifying hyperparameters in the dedicated analysis. In fact, even when practiced in an R environment, biostatisticians would perform a similar approach to realize the desired analyses. The advantage is that the user, with their own knowledge of biostatistics, does not need programming knowledge to control the input of the desired analyses and to obtain the results. This ensures that users can effectively apply statistical methods without deep R knowledge. The application led to the publication of an article [17] by being used to conduct a supplementary analysis requested by reviewers. The tool speeds up the completion of statistical analyses, useful at all stages of a project. Through the possibility of testing different variable groupings, finding or not finding initial significant results, the tool also guides towards more in-depth statistical analyses to be carried out by a biostatistician, thus saving time for all teams. After exploring their own data, physicians and researchers can arrive with more precise requests. Finally, this tool has also allowed the exploration of existing databases to assess the potential recruitment of patients in prospective studies and thus to write protocols within the framework of project calls.

The R software, open-source, is a reference tool in the field of statistics [13]. However, it requires programming knowledge to be used. The development of new R libraries in the field of data communication and web development has opened up original perspectives in the realization and automation of statistical analyses [18–20]. Knowledge of these libraries allows for the development of web applications in the form of “graphical user interfaces” (GUIs) and their easy dissemination.

However, there are other software programs with user-friendly interfaces that allow for quality statistical analyses without programming knowledge [21]. However, to name the most commonly used ones such as SPSS (https://www.ibm.com/fr-fr/spss), Stata (https://www.stata.com), or the French ones EasyMedStat (https://www.easymedstat.com/fr) and R++ (https://www.rplusplus.com/), all of these solutions are paid and thus require funding which can be substantial for institutions. The cost can thus be a major barrier to accessing such a tool. StatiCAL, being developed on R, is freely and openly accessible, providing an equivalent solution to these paid software programs and ensuring the same reliability of results [22]. Eazy R (EZR) [23, 24] and MEPHAS [25] are other comprehensive, free, and non-programming solutions, but unlike StatiCAL, they require advanced statistical knowledge. StatAid [26] better meets the needs of analyzing biomedical databases but does not allow for data manipulation and retrieval of analysis results. Jamovi (https://www.jamovi.org/download.html) and JASP (https://www.jamovi.org/) represent similar open-access tools for conducting statistical analyses. However, StatiCAL has the advantage of requiring no installation and being able to directly load databases in Excel^®^ format.

Shiny is an open-source R package that allows users to build interactive web applications directly from R, which can be deployed via a dedicated Shiny server or on a custom server. Several teams have recently used this tool to perform RNA-Seq [27] or transcriptomic [28] gene expression analyses, or to specifically analyze the performance of a diagnostic test [29]. However, StatiCAL is the first Shiny tool specifically designed to perform various statistical analyses dedicated to oncology.

This tool was created during the authors’ dedicated research time at the Centre Antoine Lacassagn. There is no monetization of this tool. It is the establishment’s intention to disseminate tools to all scientists in order to advance research. Article authors can thus cite StatiCAL in the “Statistics” section of their “Materials and Methods” sections and thus cite the reference of this article.

StatiCAL does not allow for the execution of complex analyses, and the multivariate models built by the software remain relatively basic. It is not possible to create models with interactions or to test the robustness of the models by checking underlying assumptions (regression diagnostics). We do not believe it is necessary to integrate such functions because they require more advanced biostatistics knowledge than what is typically taught in general medical and scientific studies.

The next step is to conduct a validation study of the tool. The objective will be to measure its ergonomics and usefulness by physicians and scientists with basic training in biostatistics but lacking programming knowledge. A comparison will be made between the results obtained by this group and those of senior biostatisticians using traditional tools. This study will also evaluate the relevance and reproducibility of the analyses performed as well as user satisfaction.

Certain features will be added in the next version of StatiCAL. As it can be difficult to convert data into the format supported by the application, a debugging tool and the ability to create the second sheet directly in the environment will be added. To improve the accessibility of StatiCAL, other non-proprietary formats will be supported for input files and an english version will be available.

## Conclusion

StatiCAL is a user-friendly tool, based on the R language that enables scientists without programming skills to perform statistical analyses. Its free and open-access nature represents an opportunity for everyone to gain autonomy in conducting research projects, particularly in the preliminary stages of data analysis, which helps refine requests to biostatisticians.

## Availability and requirements

Project name: StatiCAL.

Project home page: https://debds.shinyapps.io/StatiCAL/

Operating system(s): Platform independent.

Programming language: R.

Other requirements: Internet browser.

License: None.

Any restrictions to use by non-academics: None.

## Data Availability

Not applicable.

## Abbreviations

GUI: Graphical user interface
TCGA: The cancer genome atlas project
HR: Hazard ratios
